# Social Jet-Lag in Tertiary Students Following a Modern Curriculum with Few Time-Tabled Contact Hours: A Pilot Study

**DOI:** 10.3390/clockssleep1030026

**Published:** 2019-07-08

**Authors:** Cathalijn H. C. Leenaars, Mathijs F. G. Lucassen, Nedim Borger, Ellen Houben, Andries Kalsbeek

**Affiliations:** 1 Faculty of Psychology and Neuroscience, Maastricht University, 6229 ER Maastricht, The Netherlands; 2 Institute for Laboratory Animal Science, Hannover Medical School, 30625 Hannover, Germany; 3 Faculty of Veterinary Sciences, Utrecht University, 3584 CL Utrecht, The Netherlands; 4 School of Health, Wellbeing and Social Care, Walton Hall, The Open University, Milton Keynes MK7 6AA, UK; 5 Department of Psychological Medicine, Faculty of Medical and Health Sciences, University of Auckland, 1023 Auckland, New Zealand; 6 Department of Hypothalamic Integration Mechanisms, Netherlands Institute for Neuroscience (NIN), An Institute of the Royal Netherlands Academy of Arts and Sciences, Meibergdreef 47, 1105BA Amsterdam, The Netherlands; 7 Department of Endocrinology and Metabolism, Amsterdam UMC, Location AMC, University of Amsterdam (UvA), Meibergdreef 9, 1105AZ Amsterdam, The Netherlands

**Keywords:** sleep, nap, PSQI, ESS, social jet-lag, problem-based learning, students, morningness, eveningness

## Abstract

Social jet-lag (SJL) impairs academic performance, specifically for late chronotypes faced with early start times. Most modern tertiary educational systems have fewer time-tabled contact hours and consequently fewer early starts, which may limit SJL. We performed a pilot study of SJL in a convenience sample of students from Maastricht University, where problem-based learning (PBL) is implemented throughout the curricula. PBL is a modern curriculum, with only few contact hours and student-driven learning, comprising substantial amounts of self-study. Fifty-two students kept a detailed sleep diary for one week, and completed the Pittsburgh Sleep Quality Index (PSQI) and the Epworth Sleepiness Scale (ESS). Participants were divided into early and late sleepers based on a ranking of their reported sleeping times, combined with a single question on their self-reported chronotype. Late sleepers (for brevity: “Owls”; *n* = 22) had later midpoint-sleep (5:14 ± 0:11 on weekdays; 5:50 ± 0:07 on weekend days) than early sleepers (for brevity: “Larks”; *n* = 11, 3:21 ± 0:05 on weekdays; 3:41 ± 0:06 on weekend days, *F* = 10.8, *p* = 0.003). The difference between the midpoint of sleep on weekdays and weekend days was comparable for Larks and Owls (*F* = 1.5; *p* = 0.22). SJL (0:53 ± 0:06, *T* = 1.4; *p* = 0.16), total sleep duration (7:58 ± 0:08; *p* = 0.07), and PSQI score (4.7 ± 0.3, U = 137; *p* = 0.56) were comparable for Larks and Owls. Average ESS score was higher in Larks (10.7 ± 0.96) than in Owls (7.0 ± 0.72; U = 52; *p* = 0.007). Within this pilot study of students engaged in a problem-based learning curriculum, Owls have no selective disadvantage compared to Larks concerning sleep.

## 1. Introduction

We all experience differences in the preferred timing of daily activities and sleep. This “chronotype” is scientifically reflected by the midpoint of sleep on free days [1] and by several morningness-eveningness scales [2]. Social jet-lag (SJL) is the phenomenon of differences in sleep timing on work and free days, often resulting in sleep deprivation on workdays [1]. Chronotype and SJL are associated with mood, mood disorders (specifically depression), temperament, well-being, menstrual symptoms, sleep disturbances, use of stimulants and alcohol, and increased body mass index [1,3,4,5,6,7,8].

Early school (and presumably university) start times are associated with sleep deprivation and daytime sleepiness in adolescents [9]. Young adult students have been suggested to be sleepier than older participants in prior studies, probably due to mild sleep restriction as a consequence of their later chronotype [10]. Meta-analyses show that eveningness negatively impacts academic performance, and that the early schedules commonly imposed on students specifically put late chronotypes at a disadvantage [11]. A larger analysis of student logins to their learning management system shows that SJL is present in the majority of the sample, and negatively impacts academic performance [12]. However, a more recent study of secondary school students in a two-shift system shows that eveningness only hinders academic performance in students on the morning shift; it has no effect on those on the afternoon shift [13]. Yet another study highlighted that while eveningness predicts lower academic performance, SJL is not necessarily involved [14].

In recent years, many modern tertiary institutions have implemented alternative educational systems resulting in fewer contact hours and less early start-times, which might limit SJL. Maastricht University, in the South-East of the Netherlands, has implemented Problem-Based Learning (PBL) throughout their student curricula since it was founded in 1976. In this curriculum, learning is student-driven; students are primarily responsible for their own educational instruction instead of a lecturer delivering the information to the students in the traditional manner. While the exact schedule depends on the course, conventional hour-long lectures are scarce (on average less than once a week), and students meet once or twice weekly in small groups of approximately 12 students with a tutor (presence mandatory) to discuss a specific problem related to the course content. These meetings are followed by intensive self-study at the student’s discretion, which should result in 1.4 credits earned per week (based on the European Credit Transfer System, non-overlapping sequential courses, 40 h of total study time per week, 60 credits per 42-week study year). The PBL curriculum at Maastricht University thus results in relatively few contact hours compared to conventional instructor-driven curricula. A further description of a Dutch PBL curriculum is available for e.g., the Erasmus School of Law [15].

Students following a Maastricht University PBL curriculum thereby have more control over their schedules and can plan their time flexibly, logically resulting in behaviours more aligned with their chronotype than students following classic curricula. As we had good quality sleep diary data available from a previous study [16,17], we performed a pilot study on levels of SJL in Maastricht University students, as a convenience sample of students in modern curricula with limited contact hours. While we could not relate the SJL data to academic performance, we could make an informed estimate of the extent of SJL occurring within this sample.

## 2. Results

### 2.1. Demographics

Fifty-two full-time students from Maastricht University; 37 females and 15 males, completed questionnaires and sleep diaries. Forty-seven of the participants were second year psychology students following the “Research, how to do it?” course (PSY2027) at the university, for which they had to conduct, as well as participate in, research. The other 5 followed different courses. Average reported age was 22.3 years (±0.6 years, *n* = 49, range: 19–28 years). Fourteen participants (2 males) were self-reported morning types; 38 (13 males) were self-reported evening types.

Main analyses for effects of sleep timing were performed on 11 early sleepers who self-identified as morning-types (called “Larks” in the remainder of this paper, aged 22.4 ± 0.2; 2 males) and 22 late sleepers who self-identified as evening-types (called “Owls”; aged 21.8 ± 0.5; 10 males), in the remainder of the manuscript also called sleep timing types. The other 19 participants were excluded from the main analyses as they were either in the median 10% for midpoint-weekend-sleep ranking, or did not sleep consistent with their self-reported chronotype. In additional analyses we also compared self-reported chronotypes based on a single question (*n* = 14 morning types and *n* = 38 evening types) and the 25% extreme early and late sleepers (*n* = 13 each).

### 2.2. Sleep Diary Data

#### 2.2.1. Sleep Duration

Only two participants did not fully complete their sleep diary (i.e., data from one Tuesday and one Thursday were missing). For these two participants, averages were based on the remaining completed days.

During the week, students got up between 4:30 and 10:45. During the weekend, they got up between 4:15 and 11:00. Average bedtimes ranged from 22:00 to 3:48 during the week, and from 21:30 to 3:45 during the weekend. Over the seven days in which participants completed the diaries, they slept on average 7.9 h ± 0.1 per night and 8.0 h ± 0.1 per 24 h (including spontaneous daytime naps).

**Figure 1:**
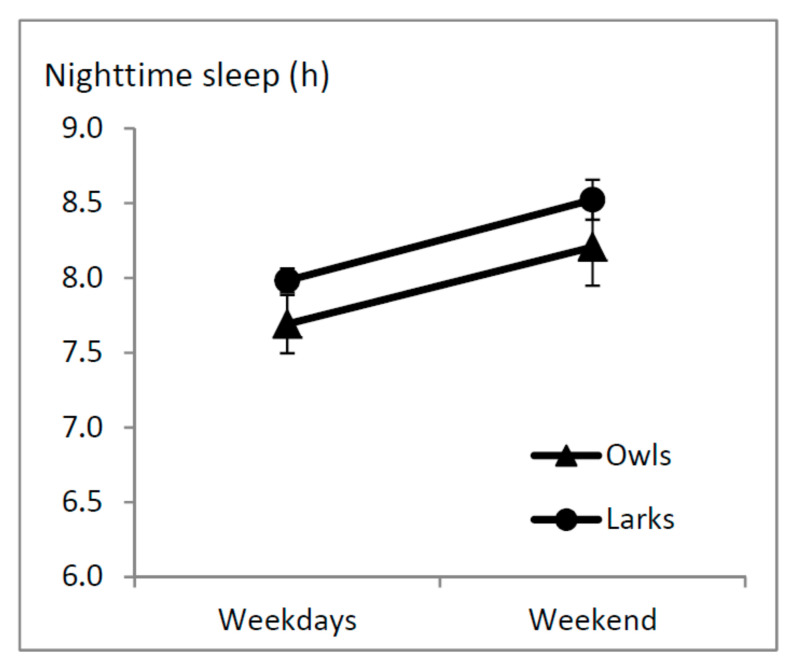
Average night-time sleep duration on weekdays and weekend days in Larks (*n* = 11) and Owls (*n* = 22) ± SEM. The effect of week vs. weekend day is significant (*p* = 0.043), the effect of Larks vs. Owls and the interaction between these two factors are not (*p* ≥ 0.36). SEM: Standard Error of the Mean, h: hour.

Average weekday night-time sleep duration was 8.0 h ± 0.1 for Larks and 7.7 h ± 0.2 for Owls. On the weekend, average night-time sleep duration was 8.5 h ± 0.1 for Larks and 8.2 h ± 0.3 for Owls (Table 1). The Analysis Of Variance (ANOVA) on night-time sleep duration showed a significant difference between weekdays and weekends (F(1,31) = 4.5; *p* = 0.043), but no difference between Larks and Owls (F(1,31) = 0.85; *p* = 0.36), or an interaction between these two factors (F(1,31) = 0.003; *p* = 0.96, Figure 1, Table 1), indicating that on average, both Owls and Larks sleep more on weekend nights than on week nights.

Average weekday Total Sleep Time (TST) was 8.0 h ± 0.1 for Larks and 7.9 h ± 0.2 for Owls. On the weekend, average TST was 8.6 h ± 0.1 for Larks and 8.3 h ± 0.3 for Owls. The ANOVA on TST showed no significant differences for any of the tested factors (Larks vs. Owls, weekdays vs. weekends and interaction), although the difference between week and weekend days approached significance (*p* = 0.070).

As the TST results (night-time sleep plus naps) were consistent with the results for night-time only sleep duration (excluding naps), data for TST are not shown.

#### 2.2.2. Midpoint Sleep

**Figure 2:**
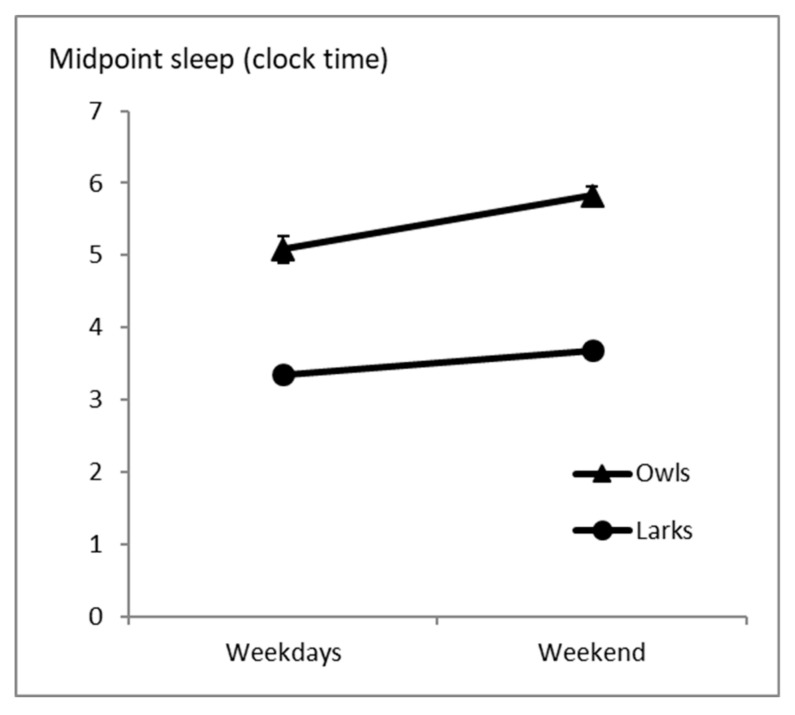
Average midpoint of sleep on weekdays and weekend days in Larks (*n* = 11) and Owls (*n* = 22) ± standard error of the mean. The effect of week vs. weekend day is significant (*p* = 0.003) as well as the effect of Larks vs. Owls (*p* < 0.001), the interaction between these two factors is not (*p* = 0.22).

During weekdays, average midpoint sleep was 3:21 (±0:05) for Larks and 5:14 (±0:11) for Owls; during the weekend, it was 3:41 (±0:06) for Larks and 5:50 (±0:07) for Owls (Table 2). The ANOVA on midpoint sleep data showed a significant difference between weekdays and weekends (F(1,31) = 10.8; *p* = 0.003), and between Larks and Owls (F(1,31) = 55.6; *p* < 0.001), but no interaction between these two factors (F(1,31) = 1.5; *p* = 0.22, Figure 2, Table 2). This indicates, as expected, that Owls have later midpoint-sleep than Larks. However, the difference between weekdays and the weekend is comparable for Larks and Owls.

#### 2.2.3. Social Jet-Lag

**Figure 3:**
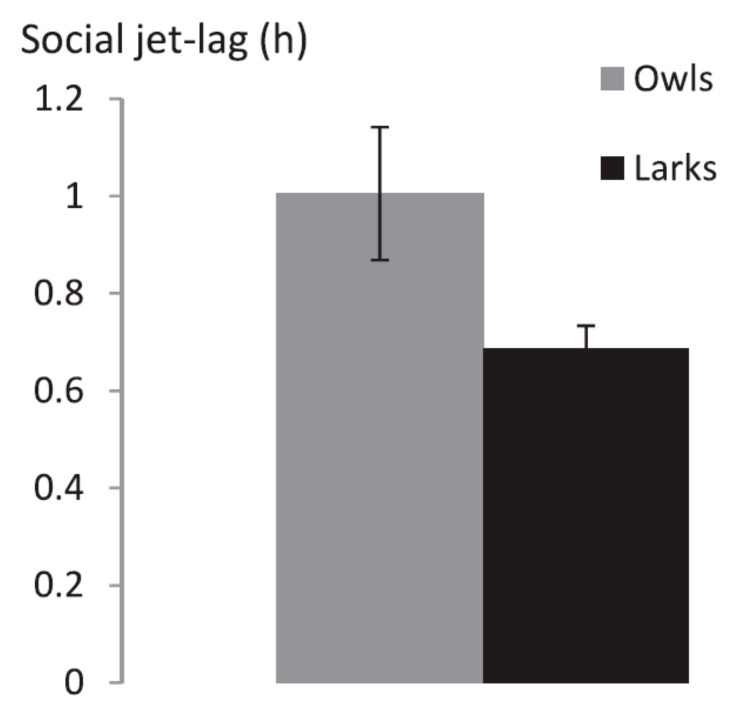
Social jet-lag (SJL) by sleep timing type (*n* = 11 Larks and *n* = 22 Owls) ± Standard Error of the Mean. There was no difference in SJL between Larks and Owls (*p* = 0.16).

Average absolute SJL (the absolute difference between midpoint sleep on week- and weekend days) was 0:53 h (±0:06) and ranged from 0 to 3:34 h. Average SJL was 0:41 ± 0:02 for Larks and 1:0 ± 0:08 for Owls. There was no difference in SJL between Larks and Owls (*T* = −1.14; *p* = 0.16, Figure 3, Table 3).

#### 2.2.4. Daytime Napping

Twenty-two students (8 males) reported spontaneous daytime napping; 14 had 1 nap during the week, 8 had multiple naps. On the night before spontaneous napping, participants reported night-time sleep duration of 8:04 h ± 0:09 h.

Four Larks reported 5 naps with an average duration of 0:45 h (±0:09); 11 Owls reported 20 naps with an average duration of 1:15 h (±0:15). The fraction of napping participants was similar for Larks and Owls (χ^2^ = 0.55; *p* = 0.46; for secondary analysis on self-reported chronotypes: χ^2^ = 0.43; *p* = 0.56; for secondary analysis on the 25% extreme sleepers: χ^2^ = 2.6; *p* = 0.11).

### 2.3. PSQI

Average Pittsburgh Sleep Quality Index (PSQI) score was 4.7 ± 0.3 (4.6 ± 0.7 for Larks and 5.1 ± 0.5 for Owls) and ranged from 0 to 10. No difference in PSQI-score was observed between Larks and Owls (U = 137; *p* = 0.56, Table 4).

A PSQI cut-off score of >5 has been suggested to have good sensitivity and specificity to identify sleep disorders [18]. Nineteen participants (five self-reported morning-types) had a PSQI-score >5. The fraction of participants with PSQI >5 could not be compared between Larks and Owls with a Chi-square test because of the small number of observations (refer to Section 4.3 for a full description of analyses). However, the analysis using the self-reported chronotypes showed that a similar fraction of participants had PSQI >5 in both groups (χ^2^ = 0.31; *p* = 0.57).

### 2.4. ESS

**Figure 4:**
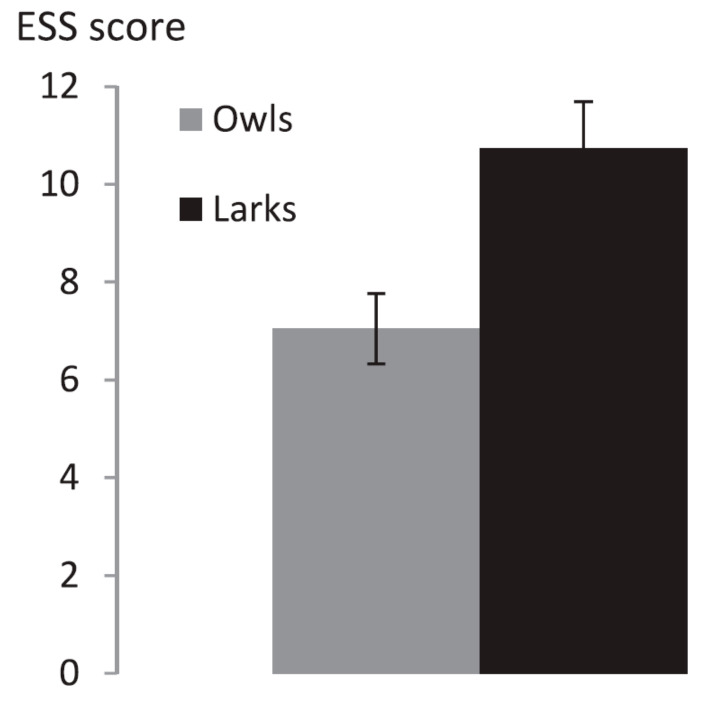
Epworth Sleepiness Scale score by sleep timing type (*n* = 11 Larks and *n* = 22 Owls) ± standard error of the mean. ESS score was higher in Larks than in Owls (U = 52; *p* = 0.007).

Average Epworth Sleepiness Scale (ESS) score was 7.6 ± 0.5 (10.7 ± 0.96 for Larks and 7.0 ± 0.72 for Owls) and ranged from 1 to 17. Average ESS score was higher in Larks than in Owls (U = 52; *p* = 0.007, Table 5, Figure 4).

The original paper describing the ESS found an ESS cut-off score of >10 in all but one participants with sleep disturbances [19]. Eleven students (five self-reported morning-types) had an ESS-score >10. The fraction of participants with ESS >10 could not be compared between Larks and Owls (or between self-reported chronotypes or between the 25% extreme early and late sleepers) because of the low number of observations.

Four students (3 Larks) had clinically relevant scores on both the PSQI (>5) and the ESS (>10).

## 3. Discussion

In this paper, we observed mild SJL in an educational system with a limited number of time-tabled contact hours.

Our sample was 71% female, reflecting the high female-to-male ratio in psychology students (e.g., [20]). Consistent with the literature (e.g., [4]), we had relatively few male Larks (*n* = 2). Because of the low number of males included in our study, we did not incorporate sex in our analyses as a predictor. For future studies, we recommend exploring sex differences, as SJL may impact women more detrimentally than in men [21].

Overall, 27% of our participants were self-reported morning types. Surprisingly, of the 23 students with the earliest weekend midpoint sleep, only 11 were self-reported morning types (Larks). This can possibly be explained by university students not being good at estimating their morningness-eveningness based on a single question. Alternatively, weekend sleep times in students could have been affected by part-time jobs which require flexibility in terms of sleep patterns. We did not determine whether participants had a part-time job, but in a study of female university students in Japan (*n* = 233), 27% had a part-time job for which they worked after 10PM [7]. We limited the effects of potential mismatches between morningness-eveningness and actual sleep patterns by focusing our main analyses on students sleeping consistent with their self-reported chronotype. For future sleep studies in university students we thus recommend including questions on part-time jobs.

A limitation of this pilot study is the absence of a validated method to determine chronotype (e.g., [2]). With the data we had available, we determined sleep timing type based on a single question combined with sleep observations over a single week. Although we did find clear differences in midpoint sleep between chronotypes, we recommend using a validated questionnaire or more objective measures (e.g., actigraphy) to determine chronotype in future studies.

Chronotype, expressed as midpoint sleep on free days corrected for sleep deprivation during the week, varies with longitude, sex, and age [4,22,23]. Concerning longitude, a geographical location more to the west within a time zone correlates with a later chronotype on average [4], consistent with light exposure (sunrise-sunset). Midpoint sleep is earlier in women than in men, but SJL is thought to be similar for both [4]. During childhood, chronotype becomes later and later until it reaches a maximum around the age of 20 years, after which it starts becoming earlier again [22].

Our participants resided in Maastricht and its surrounding areas, in the middle of the central European time zone (CET, UTC+1). A Dutch sample of high school students aged 11–18 years showed comparable midpoint sleep at 4.44 ±1.15 [24]. Roughly consistent with our sample, sleep diary data from 28 university students in Pavia (also on UTC+1, geographically more to the east) aged 19–25 years indicated midpoint sleep just after 4AM on weekdays [25]. On the weekend, when these students retired 1–2 h later, midpoint sleep was not reported. Our data are also comparable to the well-documented Munich ChronoType Questionnaire (MCTQ)-data on sleep-corrected midpoint sleep on free days, both from the western parts of Germany, and taking longitude into account, from a sample of 753 Hungarian students aged 18–35 years [4].

Consistent with our findings, the average amount of nocturnal sleep reported for various student populations is between 7 and 8 h [26], although substantially shorter nocturnal sleep periods have also been reported in previous studies of university students [20].

While our midpoint sleep times were similar to previously reported values [4,24,25], we did not replicate the difference in SJL between Larks and Owls [27]. In this pilot study, total sleep time (including daytime napping) was comparable for both sleep timing types. This indicates that within our sample, sleep-deprivation related to SJL for Owls was not as pronounced as in previous studies. While we cannot rule out other factors as no direct comparison with other samples was made, the observation of limited SJL in students on a curriculum with few contact hours is interesting and warrants further study. For future studies of modern curricula, we suggest analysing the data by days with contact hours versus days without contact hours, something that was not possible in our sample because we did not have the timetables for our participants.

A second limitation of this work is the absence of validation in terms of the health status of the participants. While the high number of participants scoring within the clinically relevant range on the PSQI (19) and ESS (11) could be reason for concern, our values are consistent with earlier studies. Previous reports on PSQI scores in students show similar results compared to ours; the clinically used score >5 has regularly been reported in over half of the participating students (e.g., [28,29,30]) and a recent review showed an average score of 6.5 in medical students [31]. Although a difference in PSQI score between chronotypes has been reported [32], this was in a sample of students also engaged in paid work and sleeping on average 6:28 h per night. Other studies showed no relationship between chronotype and PSQI score [28,33].

Average ESS score in students as reported by the developers of this questionnaire was 7.4 [34], close to our average value of 7.5. In contrast with our findings, previous work showed no effect of chronotype on ESS [32]. The direction of the effect we found is contrary to general expectations; Larks are sleepier than Owls. Further studies should elucidate if student daytime sleepiness as measured with the ESS is affected by chronotype under specific circumstances.

Another limitation of this study is the absence of objective measures of sleep. Self-reported sleep duration generally correlates poorly with more objective measures of sleep duration, such as polysomnography and actigraphy [35]. Both under- and over-reporting of sleep duration occurs, and of concern is that the discrepancies are greater in subjects with poor sleep quality, insomnia, low BMI, sleep apnoea, depression, and other forms of emotional stress [35]. This means that using self-reported measures increases the risk of confounding on sleep duration by other factors. However, we used sleep diaries and two validated sleep questionnaires instead of a single question on sleep duration. Sleep diaries are thought to be more accurate and less memory-dependent than sleep questionnaires, because they are completed daily over a longer period, shortly after waking up [36]. Besides the diaries and questionnaires, objective measures of daytime sleepiness (short versions of the sleep latency test and the maintenance of wakefulness test) after mild sleep-restriction were obtained in a subset of 15 participants. The results are presented elsewhere, but indicative of a relatively rested sample [17].

Finally, the study’s small sample size is a limitation, and so our results should thus be interpreted cautiously until they are confirmed in larger samples. We determined the sensitivity of our main (two-tailed) analyses post-hoc, based on a sample of 11 Larks and 22 Owls, α = 0.05 and β = 0.2, using GPower [37]. Our main analyses for night-time sleep could have detected within-subject effects with *f* = 0.41, between-subject effects with *f* = 0.29, and interactions with *f* = 0.29; those for midpoint sleep could have detected within-subject effects with *f* = 0.25, between-subject effects with *f* = 0.44, and interactions with *f* = 0.25, both based on the actual correlations between repeated measures. These roughly correspond to medium-sized effects within subject and for interactions, and medium to large effects between subject [38]. Still, our main analyses for SJL, ESS, and PSQI could have detected effects with an effect size of *d* = 1.1, which is considered small [38].

A major strength of this work is the quality of the data; in 52 diaries, only 2 days were missing (0.5%). A second strength of this paper is that we analyse and describe daytime napping in university students. Many studies on chronotype do not describe daytime napping at all. This is imprudent, as napping in students is common. While students in an Italian [25] and German [39] sample did not report any daytime napping, most studies addressing daytime napping have found higher napping percentages than ours (i.e., 43%), with percentages of napping ranging from 34% to 85% (reviewed by [26]). More recent work confirms high percentages of napping in undergraduate students generally (82%, [40]), and medical students (≥83.3%, [41]) and first-year psychology students (54.6%, [20]) specifically. No sex differences in napping (percentage of napping participants and nap duration) were found in 577 Mexican undergraduate students [26]. Previous work also shows that napping is less common during the weekend than during the week [26].

Because not all participants napped, it was not possible to investigate potential differences in napping behaviour between chronotypes in this pilot study; our small sample comprised only five self-reported morning types reporting six naps. Our results do, however, confirm that napping is common in university students in the Netherlands. Moreover, they indicate that nap frequency and duration may be longer in Owls than in Larks. Further research should clarify the relationship between daytime napping, chronotype, and SJL.

A third strength of this work is that we performed sensitivity analyses following our main analyses, including different subsets of participants based on slightly different criteria. The analyses of self-reported chronotype (based on the single question) were consistent with all main analyses, with one exception: the ESS score showed differences between Larks and Owls and between the 25% extreme early and late sleepers, but not between the self-reported chronotypes. Low ESS scores in the participants excluded from the other analyses may have decreased the averages and the sensitivity in this analysis. The analyses of the 25% extreme early and late sleepers, including only 13 participants in each group, deviated partially from the main analyses for night-time sleep duration and midpoint sleep where effects were observed on different combinations of main effects (week vs. weekend or Lark vs. Owl) and the interaction between them. Of note, none of the analyses showed a difference between Larks and Owls for SJL.

A fourth strength of this work is that, to the best of our knowledge, it is the first analysis of SJL within a problem-based learning curriculum. Preceding work shows that outside class periods, 93% of students reported unrestricted sleep times [4]. While we cannot confirm the actual study time tables of our sample, it seems reasonable to assume that the limited number of time-tabled contact hours in the Maastricht University system allows for sufficient sleep opportunities at personally preferred clock times during the semesters. SJL was observed, but no differences in SJL were observed between Larks and Owls. Limiting the number of contact hours in student curricula could potentially reduce or prevent relative academic disadvantages for eveningness.

## 4. Materials and Methods

### 4.1. Participants & Procedure

The study protocol was described previously [16,17]. All procedures were approved by the Ethics Committee of the Faculty of Psychology and Neuroscience of Maastricht University. All participants gave written informed consent.

The only inclusion criterion was that participants needed to be aged between 18 and 50 years (which allowed the full cohort of second-year psychology students to participate). Initially, only second-year B.Sc. psychology students were asked to participate in exchange for course credit. As the target sample size for the original study (based on *a priori* power calculation: *n* = 50 [16]) was not easily reached, recruitment was extended to include other Maastricht University students, who could participate on a purely voluntary basis.

### 4.2. Sleep Diary & Questionnaires

All questionnaires and diaries were in Dutch. All 52 participants kept an offline sleep diary for 7 days, which comprised daily bed time; time of getting up; total sleep time; number of night-time awakenings; number of daytime naps; and total nap duration. The sleep diary started with the general question “Are you a morning- or evening type?” (also in Dutch).

Participants completed the ESS [19] to assess daytime sleepiness and the PSQI [18] to assess the habitual sleep quality. The questionnaires could be completed anytime during the week that the participants kept the diaries. ESS and PSQI were programmed in Qualtrics (May 2014, Qualtrics, Provo, UT, USA. Available at: https://www.qualtrics.com).

### 4.3. Data Analysis

Data were analysed using Excel (2010, Microsoft, Redmond, WA, USA) and SPSS (IBM SPSS Statistics for Windows, version 22.0, Released 2013. IBM Corp., Armonk, NY, USA). Data are presented as mean ± Standard Error of the Mean (SEM).

For all calculations using clock times, clock times were expressed as numerical values with hours (before the comma) divided into decimals (after the comma). For calculations comprising data crossing midnight (e.g., midpoint sleep), all data were temporarily expressed in hours compared to midnight on the preceding day (e.g., 1:30 a.m. would be expressed as 25.5).

Midpoint sleep was averaged for weekdays (Sunday evening–Friday morning) and for weekend days (Friday evening–Sunday morning). Midpoint sleep was uncorrected for potential sleep deprivation. Calculations on sleep and nap durations were performed in minutes and then converted back to hours. TST was averaged for weekdays and for weekend days.

SJL was calculated by taking the absolute difference between uncorrected midpoint sleep on weekdays and weekend days [4]. While the sleep-corrected formula to determine SJL is valuable to distinguish between shifts in rhythm and sleep deprivation [42], we chose not to use it in this paper for two reasons: first, the sleep times in students are relatively irregular, and with less typical sleep patterns, the correction is not recommended; and second, we wanted to maximize the sensitivity for showing jet-lag more than distinguishing between rhythm shifts and sleep deprivation.

We performed our main analyses on “sleep timing type”; Larks were defined as self-reported morning-types within 45% extreme early weekend midpoint sleep; Owls were defined as self-reported evening-types within 45% extreme late weekend midpoint sleep. The median 10% in the midpoint-weekend-sleep ranking, and the participants not sleeping consistent with their self-reported chronotype, were excluded from analyses related to sleep timing type. We considered this combination of sleep timing and the one-question self-reported chronotype to provide the most reliable estimate of chronotype possible with the available data. This definition resulted in 11 Larks and 22 Owls taken into the main analyses.

Additional analyses were performed on all 52 participants using their self-reported chronotype (*n* = 14 self-reported morning types and *n* = 38 self-reported evening types) and on the extreme 25% of the population according to their calculated midpoint-weekend-sleep (*n* = 13 on both ends). Results from these additional analyses are provided in grey text in the tables, but not shown in the figures or otherwise described in the results, as we think that the division of participants over the chronotypes in these secondary analyses is less reliable.

Week and weekend values were compared with mixed-model analyses of variance (ANOVA), with week vs. weekend as the within-subject factor, and sleep timing type as the between-subject factor. For all ANOVA’s, a Greenhouse-Geisser correction was applied when the assumption of sphericity was violated.

Average nap duration was calculated for each napping person. The fraction of napping participants was compared between Larks and Owls with a Chi-square test.

ESS total scores were calculated as described by [19]. PSQI scores were calculated as described by [18]. ESS and PSQI scores were compared between sleep timing types with a Mann-Whitney U-test. The difference in clinically relevant scores between self-reported morning and evening types was analysed with a Chi-square test. Results from Chi-square tests were ignored if one or more of the cross-tabulation cells had an expected value of below 5.

Individual-level data for this study will not be made publicly available for protection of the privacy of the participants.

## Figures and Tables

**Table 1:** Nighttime sleep durations.

Analysis Group	Nighttime Sleep in h (SEM)		Statistics
	Weekdays	Weekend	
Larks	8.0 (0.1)	8.5 (0.1)	
Owls	7.7 (0.2)	8.2 (0.3)	
			Week vs. Wknd: F(1,31) = 4.5; *p* = 0.043 Lark vs. Owl: F(1,31) = 0.85; *p* = 0.36 Interaction: F(1,31) = 0.003; *p* = 0.96
*Self-reported morning types*	*7.9 (0.3)*	*8.4 (0.4)*	
*Self-reported evening types*	*7.9 (0.2)*	*8.1 (0.2)*	
			*Week vs. Wknd: F(1,50) = 4.2; p = 0.045* *Lark vs. Owl: F(1,50) = 0.85; p = 0.36* *Interaction: F(1,50) = 0.11; p = 0.74*
*25% extreme early sleepers*	*7.6 (0.3)*	*7.5 (0.2)*	
*25% extreme late sleepers*	*7.3 (0.2)*	*8.1 (0.4)*	
			*Week vs. Wknd: F(1,24) = 1.9; p = 0.19* *Lark vs. Owl: F(1,24) = 0.19; p = 0.67* *Interaction: F(1,24) = 3.9; p = 0.059*

Wknd: Weekend. Main analyses were performed on Larks (*n* = 11) vs. Owls (*n* = 22); early sleepers who self-identified as morning-types and late sleepers who self-identified as evening-types. Additional analyses are listed in italics. Additional analyses were performed on self-reported morning (*n* = 14) and evening (*n* = 38) types (based on a single question), and on the 25% extreme early and late sleepers (*n* = 13 each). SEM: Standard Error of the Mean, h: hour.

**Table 2:** Midpoint sleep.

Analysis Group	Midpoint Sleep; Clock Time (SEM in h)		Statistics
	Weekdays	Weekend	
Larks	3:21 (0:05)	3:41 (0:06)	
Owls	5:14 (0:11)	5:50 (0:07)	
			Week vs. Wknd: F(1,31) = 10.8; *p* = 0.003 Lark vs. Owl: (F(1,31) = 55.6; *p* < 0.001 Interaction: F(1,31) = 1.5; *p* = 0.22
*Self-reported morning types*	*3:26 (0:14)*	*4:01 (0.19)*	
*Self-reported evening types*	*4:54 (0:08)*	*5:14 (0:09)*	
			*Week vs. Wknd: F(1,50) = 7.4; p = 0.009* *Lark vs. Owl: F(1,50) = 33.3; p < 0.001* *Interaction: F(1,50) = 0.52; p = 0.47*
*25% extreme early sleepers*	*3:52 (0:22)*	*3:30 (0:16)*	
*25% extreme late sleepers*	*5:09 (0:16)*	*6:14 (0:06)*	
			*Week vs. Wknd: F(1,24) = 2.67; p = 0.12* *Lark vs. Owl: F(1,24) = 11.1; p = 0.003* *Interaction: F(1,24) = 42.8; p < 0.001*

WKND: Weekend. Main analyses were performed on Larks (*n* = 11) vs. Owls (*n* = 22); early sleepers who self-identified as morning-types and late sleepers who self-identified as evening-types. Additional analyses are listed in italics. Additional analyses were performed on self-reported morning (*n* = 14) and evening (*n* = 38) types (based on a single question) and on the 25% extreme early and late sleepers (*n* = 13 each). SEM: Standard Error of the Mean, h: hour.

**Table 3:** Social Jet-lag.

Analysis Group	Social Jet-Lag in h (SEM)	Statistics
Larks	0:41 (0:03)	*T* = −1.4; *p* = 0.16
Owls	1:00 (0:08)	
*Self-reported morning types*	*0:52 (0:12)*	*T = −0.20; p = 0.85*
*Self-reported evening types*	*0:54 (0:07)*	
*25% extreme early sleepers*	*0:52 (0:15)*	*T = −1.13; p = 0.33*
*25% extreme late sleepers*	*1:15 (0:12)*	

Main analyses were performed on Larks (*n* = 11) vs. Owls (*n* = 22); early sleepers who self-identified as morning-types and late sleepers who self-identified as evening-types. Additional analyses are listed in italics. Additional analyses were performed on self-reported morning (*n* = 14) and evening (*n* = 38) types (based on a single question), and on the 25% extreme early and late sleepers (*n* = 13 each).

**Table 4:** PSQI scores.

Analysis Group	PSQI (SEM)	Statistics
Larks	4.6 (0.7)	U = 137; *p* = 0.56
Owls	5.1 (0.5)	
*Self-reported morning types*	*9.3 (1.1)*	*U = 314; p = 0.24*
*Self-reported evening types*	*7.5 (0.6)*	
*25% extreme early sleepers*	*4.7 (0.6)*	*U = 91; p = 0.50*
*25% extreme late sleepers*	*5.0 (0.5)*	

PSQI: Pittsburgh Sleep Quality Index, SEM: Standard Error of the Mean. Main analyses were performed on Larks (*n* = 11) vs. Owls (*n* = 22); early sleepers who self-identified as morning-types and late sleepers who self-identified as evening-types. Additional analyses are listed in italics. Additional analyses were performed on self-reported morning (*n* = 14) and evening (*n* = 38) types (based on a single question) and on the 25% extreme early and late sleepers (*n* = 13 each).

**Table 5:** ESS scores.

Analysis Group	ESS (SEM)	Statistics
Larks	10.7 (1.0)	U = 52; *p* = 0.007
Owls	7.0 (0.7)	
*Self-reported morning types*	*4.1 (0.7)*	*U = 183; p = 0.11*
*Self-reported evening types*	*4.9 (0.3)*	
*25% extreme early sleepers*	*10.0 (1.1)*	*U = 39; p = 0.03*
*25% extreme late sleepers*	*6.5 (1.1)*	

ESS: Epworth Sleepiness Scale score, SEM: Standard Error of the Mean. Main analyses were performed on Larks (*n* = 11) vs. Owls (*n* = 22); early sleepers who self-identified as morning-types and late sleepers who self-identified as evening-types. Additional analyses are listed in italics. Additional analyses were performed on self-reported morning (*n* = 14) and evening (*n* = 38) types (based on a single question) and on the 25% extreme early and late sleepers (*n* = 13 each).

## Abbreviations

ANOVA	Analysis Of VAriance
ESS	Epworth Sleepiness Scale
h	hour
PBL	Problem-Based Learning
PSQI	Pittsburgh Sleep Quality Index
SEM	Standard Error of the Mean
SJL	Social Jet-Lag
TST	Total Sleep Time
